# Quality assessment of systematic reviews regarding dental implant placement on diabetic patients: an overview of systematic reviews

**DOI:** 10.4317/medoral.22955

**Published:** 2019-06-25

**Authors:** Olavo B. de Oliveira-Neto, Isabelle-Oliveira Santos, Fabiano-Timbó Barbosa, Célio-Fernando de Sousa-Rodrigues, Fernando-José C. de Lima

**Affiliations:** 1 DDS. MSc student, Department of Morphology, Anatomy Area, Piracicaba School of Dentistry, University of Campinas, Piracicaba, São Paulo, Brazil; 2 Undergraduate Medical Student, Tiradentes University (UNIT), Maceió, Alagoas, Brazil; 3 MD, PhD, Professor, Medical School, Federal University of Alagoas (UFAL), Maceió, Alagoas, Brazil; 4 MD, PhD, Professor, Department of Morphology, Human Anatomy Area, Institute of Health and Biological Sciences, Federal University of Alagoas (UFAL), Maceió, Alagoas, Brazil; 5 DDS, PhD, Professor, Department of Morphology, Human Anatomy Area, Institute of Health and Biological Sciences, Federal University of Alagoas (UFAL), Maceió, Alagoas, Brazil

## Abstract

**Background:**

Since implant placement on diabetic patients still is a controversial topic and systematic reviews are at the top of scientific evidence hierarchy, a thorough assessment of the methodological quality of these reviews must be performed to inform clinicians if their conclusions and recommendations can be followed on clinical practice. An overview of systematic reviews was performed with the purpose to assess the methodological quality of systematic reviews regarding dental implant placement on diabetic patients. In addition, we presented a synthesis of clinical outcomes about the focused theme.

**Material and Methods:**

An online search was performed on MEDLINE via PubMed, EMBASE, DARE-Cochrane, Scopus, Web of Science, LILACS, and SIGLE via Open Grey. Searches were conducted from database inception to May 2018. Systematic review articles with or without meta-analysis about the placement of dental implants on diabetic patients were included. Exclusion criteria were: articles whose primary outcome was not the survival/success rate of dental implants on diabetic patients; studies that do not relate the survival/success rate of dental implants with diabetes; duplicated papers. Methodological quality assessment was performed with AMSTAR. A descriptive synthesis of clinical outcomes was performed.

**Results:**

We identified 1.661 initial hits and eight articles were selected for overview (kappa=0.83; strong agreement). Six studies presented moderate methodological quality and two showed high methodological quality. Implant survival rate ranged from 31.8% to 100% and data from four meta-analysis showed that diabetes does not affect implant survival rate. On the other hand, data from two meta-analysis for marginal bone loss showed that diabetes statistically affects this outcome.

**Conclusions:**

Two of the eight included studies presented high methodological quality and their meta-analysis showed that implant placement on diabetic patients does not affect implant survival rate and statistically affects marginal bone loss. However, clinicians must be aware that marginal bone loss values were not clinically relevant and may not be safe to follow the conclusions and recommendations of these studies.

** Key words:**Dental implants, diabetes, systematic review, meta-analysis, survival rate.

## Introduction

Diabetes may interfere on dental implant osseointegration by suppressing osteoblastic differentiation, proliferation, and activity on this process. In addition, it may change healing and immune response due to microangiopathies caused by hyperglycemia (1-3).

Although diabetes is historically seen as a risk factor for dental implant therapy, evidences from recent studies suggest that dental implant therapy is a safe option for oral rehabilitation in diabetic patients, since it allows the patient to improve nutrition and the metabolic control, which is of the utmost importance for these patients (4,5).

Systematic reviews are at the top of scientific evidence hierarchy and their conclusions and recommendations are often followed by clinicians without a thorough judgement if these studies were properly methodologically conducted and if they present sound evidence to be safely followed on clinical practice (6,7).

Hence, this overview of systematic reviews was performed to answer the focused question: what is the methodological quality of systematic reviews regarding dental implant placement in diabetic patients?

## Material and Methods

The protocol of the present study was registered at the International Prospective Register of Systematic Reviews (PROSPERO) with the registration number CRD42018090890 (available at: http://www.crd.york.ac.uk/PROSPERO/display_record.php?ID=CRD42018090890). This overview was performed following the recommendations of the Cochrane Collaboration Handbook for Systematic Reviews (8).

We conducted an overview of systematic reviews with the aim to determine the methodological quality of systematic reviews regarding dental implant placement on diabetic patients. In addition, we presented a synthesis of clinical outcomes in regard to the focused theme.

Since this study is an overview of systematic reviews, whose sample is systematic review articles, it was not submitted to an ethics committee and it does not require an informed consent.

The present study included systematic review articles with or without meta-analysis about the placement of dental implants on diabetic patients. Exclusion criteria were: articles whose primary outcome was not the survival/success rate of dental implants on diabetic patients; studies that do not relate the survival/success rate of dental implants with diabetes; duplicated papers (papers found on more than one database). This systematic review did not establish restrictions regarding language or status of publications and was not influenced by persons or institutions.

The search was performed on MEDLINE via PubMed, EMBASE, DARE-Cochrane, Scopus, Web of Science, LILACS, and SIGLE via Open Grey. Non-peer-reviewed literature (gray literature) was sought on SIGLE via Open Grey. Searches were conducted from database inception to May 2018. A comprehensive search strategy was set individually for each database, as follows.

- MEDLINE via PubMed: ((((((((((((((dental implants) OR implants, dental) OR dental implant) OR implant, dental) OR dental prostheses, surgical) OR dental prosthesis, surgical) OR surgical dental prosthesis) OR surgical dental prosthesis) OR prostheses, surgical dental) OR prosthesis, surgical dental))))) AND (((((((((((((((((diabetes mellitus) OR diabetes mellitus, type 2) OR diabetes mellitus, noninsulin-dependent) OR diabetes mellitus, non insulin dependent) OR diabetes mellitus, non-insulin-dependent) OR non-insulin-dependent diabetes mellitus) OR diabetes mellitus, noninsulin dependent) OR type 2 diabetes mellitus) OR type 2 diabetes) OR diabetes, type 2) OR diabetes mellitus, type 1) OR diabetes mellitus, insulin-dependent) OR diabetes mellitus, insulin dependent) OR insulin-dependent diabetes mellitus) OR type 1 diabetes mellitus) OR type 1 diabetes) OR diabetes, type 1)

- EMBASE: (‘diabetes mellitus’/exp OR ‘diabetes’ OR ‘diabetes mellitus’ OR ‘diabetic’) AND (‘tooth implantation’/exp OR ‘dental implantation’ OR ‘dental implantation, endosseous’ OR ‘dental implantation, endosseous, endodontic’ OR ‘dental implantation, subperiosteal’ OR ‘immediate dental implant loading’ OR ‘tooth implantation’ OR ‘tooth implant’/exp OR ‘dental implant’ OR ‘dental implants’ OR ‘endosseous dental implant’ OR ‘implant, teeth’ OR ‘implant, tooth’ OR ‘implants, teeth’ OR ‘implants, tooth’ OR ‘teeth implant’ OR ‘teeth implants’ OR ‘tooth implant’ OR ‘tooth implants’)

- DARE-Cochrane: MeSH descriptor: [Dental Implants] explode all trees or tooth implant:ti,ab,kw (Word variations have been searched) or tooth implantation:ti,ab,kw (Word variations have been searched) or diabetes:ti,ab,kw (Word variations have been searched)

- Web of Science: ts=(dental implant OR tooth implant OR tooth implantation) or ts=(diabetes OR diabetes mellitus)

- SCOPUS: ( TITLE-ABS-KEY ( “dental implant” ) OR TITLE-ABS-KEY ( “tooth implant” ) OR TITLE-ABS-KEY ( “tooth implantation” ) AND TITLE-ABS-KEY ( “diabetes” ) OR TITLE-ABS-KEY ( “diabetes mellitus” ) )

- LILACS: dental implant OR tooth implant OR tooth implantation OR implantação dentária AND Diabetes

- SIGLE via Open Gray: “dental implants” OR “tooth implant” OR “tooth implantation” AND “diabetes” 

Considering eligibility criteria, the search for eligible studies was conducted by two reviewers (O.B. and I.O.), which initially screened titles and/or abstracts in order to select potential eligible studies to be read fully. Final inclusion was then established, and references of selected papers were also hand-searched. These reviewers tried to establish a consensus in cases of disagreements a third, and more experienced, reviewer (F.T.) was consulted in situations where any disagreement persisted.(9,10) 

Once papers were included for overview, a methodological quality assessment was performed by the same reviewers (O.B. and I.O.) using AMSTAR. This tool is composed of an 11-item questionnaire that focused on potential sources of bias regarding the review process and determines a final score that ranges from 0 to 11 and represents the methodological quality of the review as high, moderate, or low (11-13). Specific items from AMSTAR and its classification system are described on  Table 1.

**Table 1:**
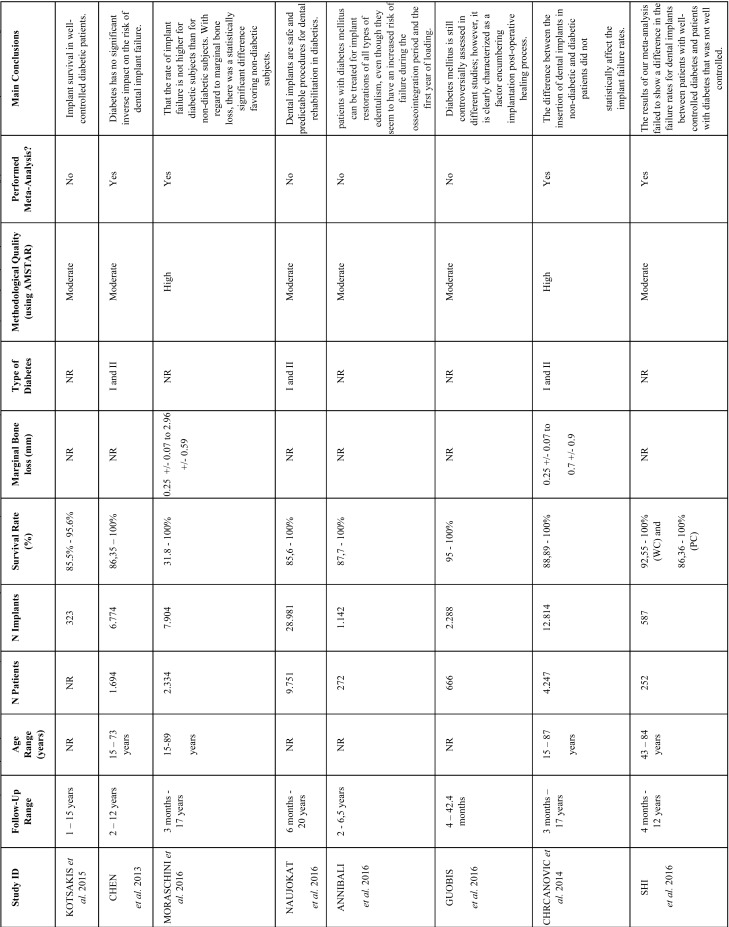
Characteristics of included studies. NR= not reported; WCD= well controlled diabetes; PCD= poorly controlled diabetes; CCT = controlled clinical trial; RCT = randomized clinical trial; PC= prospective cohort; RC = retrospective cohort; CC= case-control; CS = case series; Prosp = prospective study; Retro= retrospective study.

The primary outcome of the present overview was the methodological quality of included systematic reviews. Secondary outcomes were: implant survival/success rate, marginal bone loss, follow-up time, and patient’s age. Type of diabetes, number of patients and number of implants were complementary outcomes.

-Data analysis

An overview of systematic reviews does not require sample size calculation and, hence, it was not performed. Cohen’s kappa analysis was performed to measure the level of agreement between reviewers on the selection of eligible studies.

Methodological quality assessment with AMSTAR (primary outcome) was presented in a score from 0 to 11 and classified as of high, moderate, or low methodological quality.

Secondary outcomes were described as follows: implant survival/success rate as percentages, marginal bone loss in millimeters, follow-up range and patient’s age range in years; and type of diabetes was described according to author’s report.

Effect measures of systematic reviews that performed meta-analysis regarding implant failure and marginal bone loss were described, respectively, as relative risk or mean difference, each with its confidence interval and with values calculated for heterogeneity.

## Results

-Search and selection process

**Figure 1:**
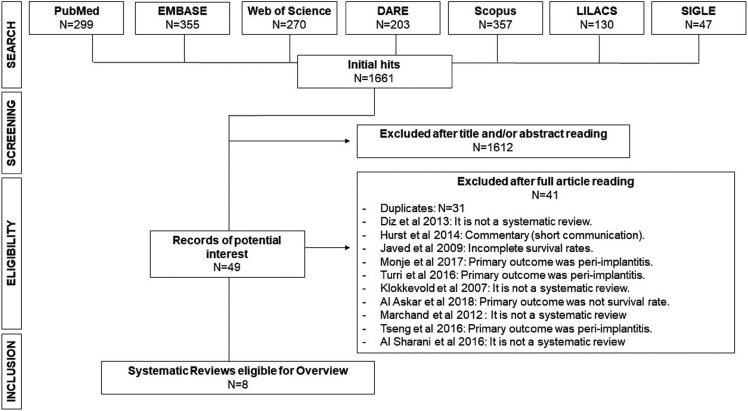
Flow chart showing steps performed to select eligible studies for overview.

We identified 1.661 initial hits, which were distributed as follows: MEDLINE via PubMed= 299; EMBASE= 355; Web of Science= 270; DARE-Cochrane= 203; Scopus= 357; LILACS= 130; and SIGLE via Open Gray= 47. After title and/or abstract reading, 1.612 papers were excluded. Then, the 49 remaining publications were read fully, and 41 articles were excluded. Finally, eight articles were selected for overview (4,5,14-19). Figure 1 shows a synthesis of search and selection process as well as excluded papers and reasons for exclusions (20-28).

Cohen’s Kappa statistics was calculated and showed an inter-reviewer agreement of 0.83, (strong agreement).

-Methodological Quality Assessment

Of the eight selected papers, two were classified as of “high” methodological quality (Moraschini *et al.* and Chrcanovic *et al.*) (5,18) and six were of “moderate” methodological quality. Scores ranged from 5/11 (Kotsakis *et al.*) (14) from 10/11 (Moraschini et al. and Chrcanovic *et al.*).

Question 1 from AMSTAR received the lowest number of “Yes” answers (12,5%) and questions 5, 6, and 7 from AMSTAR received the highest number of “Yes” answers (100% each). Overall positive answers (Yes answers) comprised 63,6% of results. Individual scores from each paper as well as specific items from AMSTAR and its classification system are described on  Table 2.

**Table 2:**
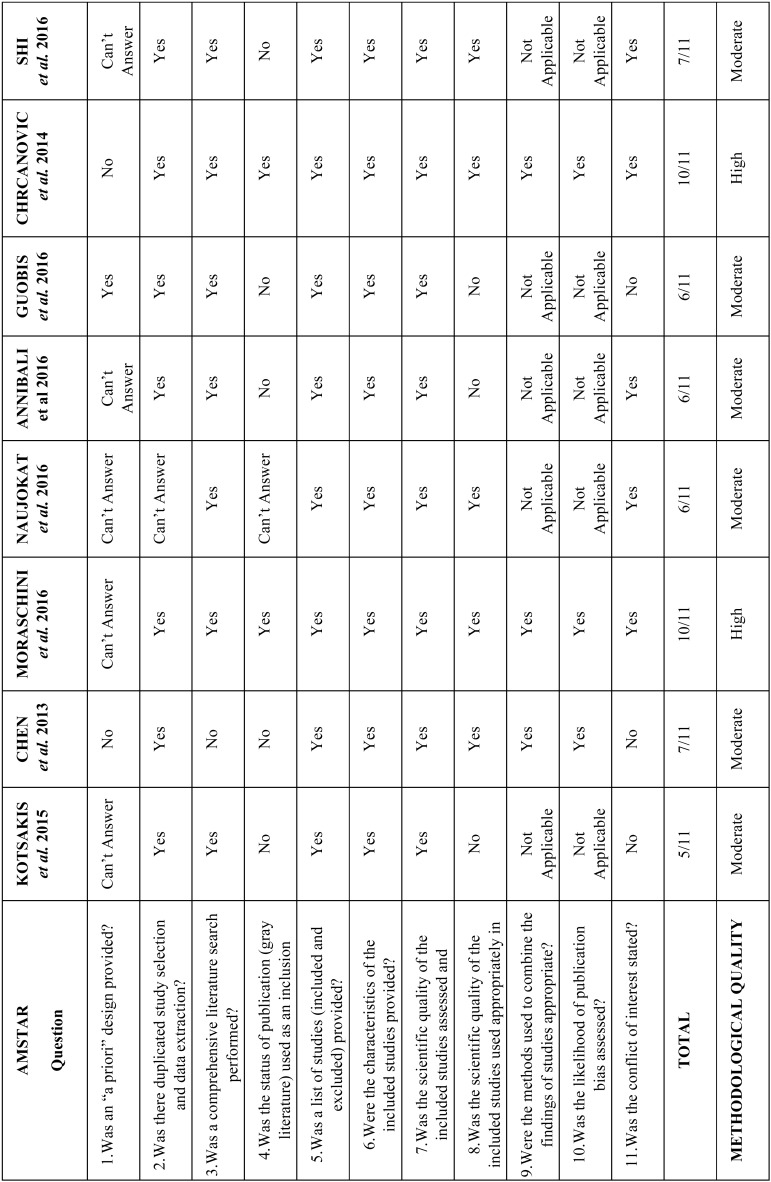
Methodological quality assessment of included studies using AMSTAR. The methodological quality was determined by the number of “Yes” answers and each study was classified as of High (9-11 “yes” answers), Moderate (5-8 “yes” answers), or Low (0-4 “Yes” answers). “Can’t answer” was chosen when the item was not reported by the authors, and “not applicable” was chosen when the item did not match the study type (e.g. the item could only be properly answered if a meta-analysis was performed).

-Implant survival /success rate

Considering all included studies, implant survival rate ranged from 31.8% to 100%. However, Chen *et al.* (4) (relative risk=0.90; 95% confidence interval: 0.62 – 1.32; 3 studies; 6.774 implants), Moraschini *et al.* (5) (relative risk=1.56; 95% confidence interval: 0.62 – 3.91; 7.904 implants), Chrcanovic *et al.* (18) (relative risk=1.07; 95% confidence interval: 0.80 – 1.44; 12.814 implants), and Shi *et al.* (19) (relative risk=0.62; 95% confidence interval: 0.22 – 1.70; 587 implants) performed meta-analysis for implant survival /success rate and did not present statistically significant results for this outcome, which showed that implant placement on diabetic patients does not affect implant survival rate ( Table 3).

**Table 3:**
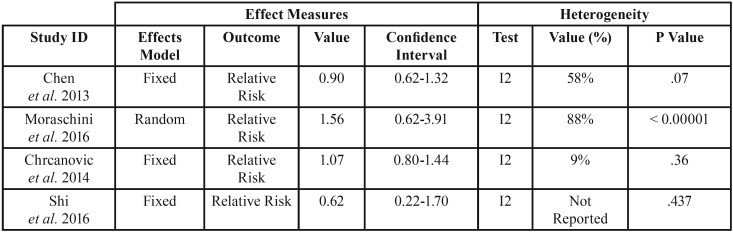
Results from meta-analysis regarding implant failure.

-Marginal bone loss

Chrcanovic *et al.* (mean difference= 0.20; 95% confidence interval: 0.08-0.31) and Moraschini *et al.* (mean difference= 0.18; 95% confidence interval: 0.14-0.21) reported data regarding marginal bone loss and performed meta-analysis for this outcome, which showed that implant placement on diabetic patients affects marginal bone loss, with statistically significant results ( Table 4).

**Table 4:**
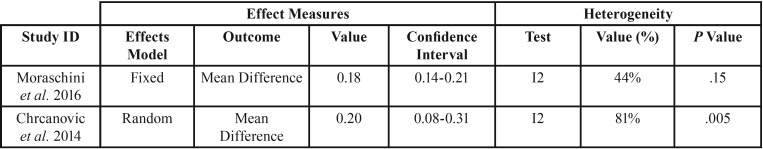
Results from meta-analysis regarding marginal bone loss.

-Follow-up range and patient’s age range

One can see that five studies assessed patients with less than one year of follow-up, and the other three studies assessed patients with at least one or two years of follow-up. Four studies reported patient’s age, which ranged from 15 to 89 years.

## Discussion

An overview of eight systematic reviews regarding the placement of dental implants on diabetic patients was conducted. In addition, we presented a synthesis of clinical outcomes about the focused theme. Our assessment with the AMSTAR tool determined that only two of the eight eligible reviews were classified as of high methodological quality and six were classified as of moderate methodological quality.

Although the studies of Moraschini et al. and Chrcanovic et al. (5,18) were classified as of “high” methodological quality and received the highest score between included studies (10/11), they did not mention if a protocol was developed a priori (question 1 from AMSTAR), which makes us question if the authors properly planned their reviews or if they changed methodology along the way.

The findings from Chen *et al.* (4) (relative risk=0.90; 95% confidence interval: 0.62 – 1.32; 3 studies; 6.774 implants), Moraschini *et al.* (5) (relative risk=1.56; 95% confidence interval: 0.62 – 3.91; 7.904 implants), Chrcanovic *et al.* (18) (relative risk=1.07; 95% confidence interval: 0.80 – 1.44; 12.814 implants), and Shi *et al.* (19) (relative risk=0.62; 95% confidence interval: 0.22 – 1.70; 587 implants) indicate that diabetes does not affect implant survival rate. Nevertheless, these results must be carefully reviewed because two of these studies were classified as of moderate methodological quality, two presented clinical biases, and one performed meta-analysis of studies with a considerable amount of heterogeneity (I2=88%; *p* <0.00001). One must highlight that the results from Shi *et al.* (19) considered patients with well controlled diabetes and patients with poorly controlled diabetes, and the pooled meta-analysis showed that diabetes does not affect survival rate, regardless of glycemic control. It’s also worth mentioning that Moraschini *et al.* (5) reported values of 31.8% for implant survival rate, which is considered as a very low rate and, even so, their meta-analysis also showed that diabetes does not affect implant survival rate. This meta-analysis identified on the funnel plot the study that caused such a low survival rate and did not go any further to reanalyze results without this study and/or to discuss what caused this outlier result. Thus, we considered that this may had strongly affected this meta-analysis results for implant survival rate.

Additionally, results from Moraschini *et al.* (5) (mean difference= 0.18; 95% confidence interval: 0.14-0.21) and Chrcanovic *et al.* (18) (mean difference= 0.20; 95% confidence interval: 0.08-0.31) indicate that diabetes significantly affects marginal bone loss. One must highlight that, despite the statistical significance of these results, they were not clinically relevant, since a loss of one fifth of a millimeter is a very low measure and may have occurred due to the surgical technique. Additionally, besides clinical biases, these results were statistically combined with studies of moderate and high heterogeneity, respectively ( Table 4).

Regarding follow-up and patients’ age, a short and inadequate follow-up of less than one year was identified on several studies and the authors assessed patients still at a young age (15 years old). (5,15,17-19) A short follow-up is a strong clinical bias because a dental implant may be lost on an earlier or on a later period, thus won’t be possible to identify implant loss after the short follow-up. Since patients at a young age are on the stage of bone development, the placement of dental implants on these patients are also considered a source of bias. These flaws may have acted as confounding factors and may have influenced the results of several included reviews, including the ones that were assessed as of high methodological quality.

An overview is a tertiary study that assesses secondary studies, which by their turn, gather data from primary publications. (6,7) Hence, the main limitation of the present study was the distance between tertiary and primary data, since an overview’s conclusions are drawn from secondary studies. Nevertheless, it becomes important to assess these secondary studies since they are often sought for clinical decision making and may be inadequately followed.

To the best of the authors’ knowledge, this is the first overview of systematic reviews regarding dental implant placement on diabetic patients. Going forward, future systematic reviews must combine a high methodological quality with an unbiased selection of included studies with more precise eligibility criteria, also properly considering an adequate follow-up time and avoiding young and elderly patients (age extremes). Also, clinical parameters that may indicate an early possibility of implant failure, such as bleeding on probing and presence of gingival infection must be considered for primary and secondary studies. This set of improvements will provide robust scientific evidence for clinical practice.

Hence, the present overview of systematic reviews concluded the following:

- Two of the eight included studies presented high methodological quality and their meta-analysis showed that dental implant placement on diabetic patients does not affect implant survival and statistically affects marginal bone loss;

- Clinicians must be aware that marginal bone loss values were not clinically relevant and may not be safe to follow the conclusions and recommendations of these studies.
